# Real-world data on vitamin D supplementation and its impacts in systemic lupus erythematosus: Cross-sectional analysis of a lupus registry of nationwide institutions (LUNA)

**DOI:** 10.1371/journal.pone.0270569

**Published:** 2022-06-29

**Authors:** Keigo Hayashi, Ken-Ei Sada, Yosuke Asano, Yu Katayama, Keiji Ohashi, Michiko Morishita, Yoshia Miyawaki, Haruki Watanabe, Takayuki Katsuyama, Mariko Narazaki, Yoshinori Matsumoto, Nobuyuki Yajima, Ryusuke Yoshimi, Yasuhiro Shimojima, Shigeru Ohno, Hiroshi Kajiyama, Kunihiro Ichinose, Shuzo Sato, Michio Fujiwara, Jun Wada

**Affiliations:** 1 Department of Nephrology, Rheumatology, Endocrinology and Metabolism, Okayama University Graduate School of Medicine, Dentistry and Pharmaceutical Sciences, Okayama, Japan; 2 Department of Clinical Epidemiology, Kochi Medical School, Nankoku, Japan; 3 Department of Medicine, Division of Rheumatology, Showa University School of Medicine, Tokyo, Japan; 4 Center for Innovative Research for Communities and Clinical Excellence, Fukushima Medical University, Fukushima, Japan; 5 Department of Stem Cell and Immune Regulation, Yokohama City University Graduate School of Medicine, Yokohama, Japan; 6 Department of Medicine (Neurology and Rheumatology), Shinshu University School of Medicine, Matsumoto, Japan; 7 Center for Rheumatic Diseases, Yokohama City University Medical Center, Yokohama, Japan; 8 Department of Rheumatology and Applied Immunology, Faculty of Medicine, Saitama Medical University, Saitama, Japan; 9 Department of Immunology and Rheumatology, Advanced Preventive Medical Sciences, Nagasaki University Graduate School of Biomedical Sciences, Nagasaki, Japan; 10 Department of Rheumatology, Fukushima Medical University School of Medicine, Fukushima, Japan; 11 Department of Rheumatology, Yokohama Rosai Hospital, Yokohama, Japan; Nippon Medical School, JAPAN

## Abstract

**Background:**

Although vitamin D concentration is reportedly associated with the pathogenesis and pathology of systemic lupus erythematosus (SLE), benefits of vitamin D supplementation in SLE patients have not been elucidated, to our knowledge. We investigated the clinical impacts of vitamin D supplementation in SLE.

**Methods:**

A cross-sectional analysis was performed using data from a lupus registry of nationwide institutions. We evaluated vitamin D supplementation status associated with disease-related Systemic Lupus International Collaborating Clinics/American College of Rheumatology Damage Index (SDI) as a parameter of long-term disease activity control.

**Results:**

Of the enrolled 870 patients (mean age: 45 years, mean disease duration: 153 months), 426 (49%) received vitamin D supplementation. Patients with vitamin D supplementation were younger (43.2 vs 47.5 years, *P* < 0.0001), received higher doses of prednisolone (7.6 vs 6.8 mg/day, *P* = 0.002), and showed higher estimated glomerular filtration rates (79.3 vs 75.3 mL/min/1.73m^2^, *P* = 0.02) than those without supplementation. Disease-related SDI (0.73 ± 1.12 vs 0.73 ± 1.10, P = 0.75), total SDI, and SLE Disease Activity Index (SLEDAI) did not significantly differ between patients receiving and not receiving vitamin D supplementation. Even after excluding 136 patients who were highly recommended vitamin D supplementation (with age ≥ 75 years, history of bone fracture or avascular necrosis, denosumab use, and end-stage renal failure), disease-related SDI, total SDI, and SLEDAI did not significantly differ between the two groups.

**Conclusions:**

Even with a possible Vitamin D deficiency and a high risk of bone fractures in SLE patients, only half of our cohort received its supplementation. The effect of vitamin D supplementation for disease activity control was not observed.

## Introduction

Vitamin D deficiency plays a role in both the pathogenesis and pathology of systemic lupus erythematosus (SLE). SLE patients exhibit a higher prevalence of vitamin D deficiency [1, 2], and 1,25-dihydroxy vitamin D plays a preventive role in SLE incidence by regulating type 1 interferon and antibody production by inhibiting dendritic cell differentiation and T and B cell proliferation [3–5]. NK-cell immunity is as well critical for viral and immune mediated diseases and its dysregulation as a consequence of vitamin D deficiency could worsen disease activity in SLE also influence on disease activities by dysregulating NK-cell immunity [6–8]. Vitamin D deficiency also influences the microbiome patterns which are associated with autoimmune diseases such as SLE [9]. Vitamin D concentration shows an inverse correlation with anti-double-stranded DNA (anti-dsDNA) antibody titres [10] and disease activity [10–13] in SLE patients.

Pathology of SLE has been reported to be strongly associated with vitamin D deficiency; however, the positive effects of vitamin D supplementation on disease activity in SLE patients have not yet been elucidated, to our knowledge [14]. Clinical guidelines [15–18] have recommended vitamin D supplementation only for osteoporosis treatment in SLE patients [18]. Considering that glucocorticoids and other immunosuppressants have been reported to control disease activity enough in SLE patients, it is difficult to evaluate the relationship between vitamin D supplementation and short-term disease activity control in SLE patients [14]. The Systemic Lupus International Collaborating Clinics/American College of Rheumatology (SLICC/ACR) Damage Index (SDI) is used to assess disease- and treatment-related damage accumulation during the clinical course of SLE [19–21]. Using disease-related SDI as a marker for the accumulative consequences of long-term disease activity control in SLE patients [22], we evaluated the clinical impact of vitamin D supplementation on SLE patients. Since SDI. Since organ damage in SLE accumulates over a long period of time, with a reported change in SDI of less than 1 in 10 years [23], we evaluated the outcome cross-sectionally for our cohort with a relatively long disease duration, averaging more than ten years.

The objective of this study was to describe vitamin D supplementation status in daily clinical practice and evaluate the relationship between vitamin D supplementation and disease-related damage accumulated as a result of disease activity in SLE patients.

## Materials and methods

### Database

Established in 2016, the lupus registry of nationwide institutions (LUNA) is a nationwide multicentre observational cohort database of SLE patients from 9 referral institutions in Japan. All patients were over 20 years of age and had scores of ≥4 according to the ACR classification criteria for SLE [24]. Baseline data obtained at registration included demographic information, date of disease onset, underlying comorbidities, smoking and drinking habits, medical history, reproductive history, and blood pressure. Laboratory data obtained at registration included results of complete blood count analysis, biochemical examination, urinalysis, complement level analysis, anti-dsDNA antibody titre analysis, and antiphospholipid antibody (lupus anticoagulant and anticardiolipin-β2-glycoprotein I complex antibodies) analysis. Disease activity and damage were evaluated using the SLE Disease Activity Index (SLEDAI) [24] and SDI [25], respectively. Data regarding current and previous treatments for SLE, such as glucocorticoid dosage and concomitant immunosuppressant use, and treatments for comorbidities including osteoporosis, such as calcium and vitamin D supplementation and bisphosphonate, denosumab, and teriparatide use, were also collected.

### Patient selection

In the present study, all inpatients and outpatients registered in the LUNA registry up until August 2019 were enrolled. Vitamin D supplementation status was evaluated in all the registered patients except those who were currently pregnant or had malignancies.

For sensitivity analysis, patients who were highly recommended vitamin D supplementation were excluded using the following criteria: (1) age ≥ 75 years at SLE diagnosis (high prevalence of osteoporosis), (2) history of bone fracture or avascular necrosis (requiring vitamin D supplementation for secondary prevention of bone disease), (3) use of denosumab (requiring concomitant vitamin D supplementation), and (4) presence of end-stage renal failure (requiring vitamin D supplementation for chronic kidney disease-mineral and bone disorder). Patients with end-stage renal failure were defined as those with irreversible levels of estimated glomerular filtration rates (eGFRs) <15 mL/min/1.73m^2^ or those requiring dialysis irrespective of the eGFR [26]. We have no data on past status or duration of vitamin D supplement. Therefore, we also performed additional analysis including patients only with duration ≥ 60 months to excluded those who might take vitamin D supplement only for short periods. Another analysis was performed excluding patients without prednisolone for dealing with possible effects of prednisolone use on vitamin D levels.

### Outcome measures

The primary outcome measure in this study was disease-related SDI and the respective domains constituting total SDI. Disease-related SDI comprised SDI items (neuropsychiatric, renal, pulmonary, cardiovascular, peripheral vascular, gastrointestinal, and skin involvements, retinal change or optic atrophy, muscle atrophy/weakness, arthritis, osteomyelitis, premature gonadal failure, and malignancy), associated with SLE after excluding items mainly associated with glucocorticoid-use-related adverse effects (avascular osteonecrosis, osteoporotic fractures, diabetes mellitus, and cataracts), as defined [27] and validated [28] by previous studies. Secondary outcome measures were total SDI and SLEDAI at registration.

### Statistical analysis

First, we described vitamin D supplementation status and associated patients’ characteristics. We also described the prevalence of bone diseases and other concomitant treatments for osteoporosis in all the enrolled patients. Next, we compared outcomes between patients with and those without vitamin D supplementation. For sensitivity analysis, we excluded patients who were highly recommended vitamin D supplementation and then compared outcomes between patients with and those without vitamin D supplementation. Continuous variables were compared using the Mann-Whitney *U* test or Welch’s *t*-test, depending on data distribution between the two groups. Categorical variables were compared using the chi-squared test or Fisher’s direct probability test, as appropriate. The odds ratios for one or more disease-related SDI items were calculated for the respective domains constituting total SDI and were compared between patients receiving and not receiving vitamin D supplementation. For outcome variables that were significant in the univariate analysis, multivariate logistic regression analysis was performed to adjust for age, sex and PSL dose as possible confounding factors.

Missing data were seen on disease durations and prednisolone (PSL) doses and were complemented by multivariate normal imputation using the least squares method.

Clinical characteristics are presented as mean ± standard deviation (SD) for continuous variables and patient number (%) for categorical variables. *P* values < 0.05 were considered statistically significant. All statistical analyses were performed using the JMP statistical software package for Windows, version 12.2.0 (SAS Institute Inc., Cary, NC, USA).

### Ethics approval and consent

This study was conducted according to the Declaration of Helsinki and the ethical guidelines for epidemiologic research in Japan. The study protocol was approved by the ethics committee of Okayama University Graduate School of Medicine, Dentistry and Pharmaceutical Sciences (authorisation number: Ken1909-025). All patients provided written informed consent to participate in the registry and gave permission to have their data published.

## Results

### Patient characteristics

Of the 929 patients registered in the LUNA study, 17 were excluded owing to a lack of data, 3 were excluded owing to current pregnancies, and 39 were excluded owing to current malignancies. In the 870 enrolled patients with a mean ± SD age of 45 ± 14 years and a mean disease duration of 153 ±121 months, the mean SLEDAI score was 5.3 ± 4.8 and the mean total SDI score was 1.1 ± 1.5. The mean PSL dose at registration was 7.2 ± 6.5 mg/day, and the mean previous maximum PSL dose was 40 ± 21 mg/day. Hydroxychloroquine was administered to 225 (26%) patients. With respect to immunosuppressants, azathioprine was administered to 99 (11%) patients, mycophenolate mofetil was administered to 112 (13%) patients, and tacrolimus was administered to 296 (34%) patients.

### Vitamin D supplementation and osteoporosis treatment

Of the 870 enrolled patients, 426 (49%) received vitamin D supplementation. Characteristics of patients receiving (n = 426) and not receiving (n = 444) vitamin D supplementation are shown in Table 1. Patients receiving vitamin D supplementation were younger (43.2 ± 14.6 vs 47.5 ± 14.5 years, *P* < 0.0001), had higher PSL doses (7.6 ± 6.2 vs 6.8 ± 6.7 mg/day, *P* = 0.002), showed higher eGFRs (79.3 ± 26.1 vs 75.3 ± 24.7 mL/min/1.73m^2^, *P* = 0.02), and concomitantly used bisphosphonate less frequently (38% vs 46%, *P* = 0.02) than those not receiving vitamin D supplementation.

**Table 1:** Comparison of patient characteristics.

Characteristics	Primary analysis (n = 870)			Sensitivity analysis (n = 731)		
	Vit. D (+) (n = 426)	Vit. D (-) (n = 444)	*P* value	Vit. D (+) (n = 358)	Vit. D (-) (n = 376)	*P* value
Age, years	43.2 ± 14.6	47.5 ± 14.5	<0.0001	41.0 ± 13.1	46.1 ± 13.3	<0.0001
Disease duration, months	149 ± 116	157 ± 126	0.33	136 ± 108	149 ± 125	0.14
Number of female patients, n (%)	385 (90)	385 (86)	0.09	329 (92)	333 (89)	0.13
Mean SLEDAI, n (%)	5.5 ± 5.1	5.2 ± 4.5	0.62	5.5 ± 4.9	5.2 ± 4.5	0.40
Mean eGFR, mL/min/1.73 m^2^	79.3 ± 26.1	75.3 ± 24.7	0.02	81.0 ± 25.5	77.1 ± 23.9	0.03
PSL dose at registration, mg/day	7.6 ± 6.2	6.8 ± 6.7	0.002	7.8 ± 5.8	6.9 ± 7.1	0.0006
Max. PSL dose after diagnosis, mg/day	40.9 ± 16.3	38.5 ± 18.8	0.05	40.7 ± 16.0	37.6 ± 18.8	0.02
Bisphosphonate use, n (%)	163 (38)	206 (46)	0.02	139 (39)	176 (47)	0.03

Vit., vitamin; SLEDAI, Systemic Lupus Erythematosus Disease Activity Index; eGFR, estimated glomerular filtration rate; PSL, prednisolone; max., maximum.

In this study, 95 (11%) patients experienced fractures or avascular necrosis and 657 (76%) received at least one treatment for osteoporosis; 426 (49%) received vitamin D supplementation, 36 (4%) received calcium supplementation, 369 (42%) received bisphosphonates, 14 (2%) received teriparatide, 16 (2%) received denosumab, and 4 (0.005%) received selective oestrogen receptor modulators. Of the 426 patients with vitamin D supplementation, 49 (12%) experienced fractures or avascular necrosis. Patients with a history of fracture or avascular necrosis were older (45 ± 15 vs 51 ±15 years, *P* < 0.0001) and received denosumab (7% vs 1%, *P* < 0.0001) or teriparatide (7% vs 1%, *P* < 0.0001) more frequently than those without a history of fracture or avascular necrosis. There were no differences in vitamin D supplementation (52% vs 49%, *P* = 0.59) and bisphosphonate prescription (40% vs 43%, *P* = 0.61) between patients with and without a history of fracture or avascular necrosis (S1 Table).

### Outcomes related to vitamin D supplementation

**Fig 1:**
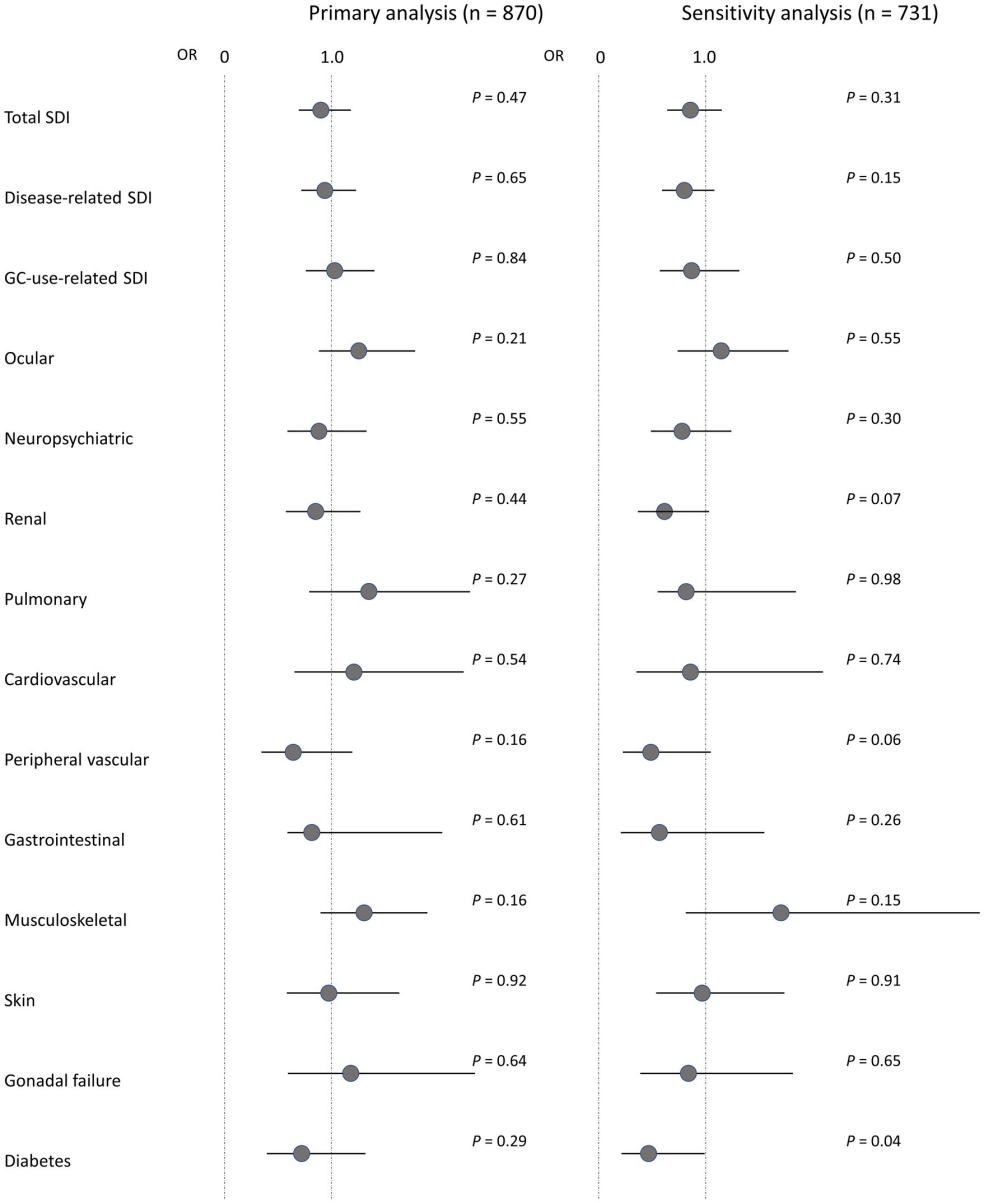
ORs for SDI between patients receiving and not receiving vitamin D supplementation.

No significant differences in disease-related SDI and the respective domains of total SDI were observed between patients receiving and not receiving vitamin D supplementation (disease-related SDI: 0.73 ± 1.12 vs 0.73 ± 1.10, *P* = 0.75) (S2 Table). For sensitivity analysis, 136 patients with age ≥ 75 years, a history of bone fracture or avascular necrosis, and end-stage renal disease were excluded. Characteristics of the included patients receiving (n = 358) and not receiving (n = 376) vitamin D supplementation are shown in Table 1. Even in the included patients, no significant differences in disease-related SDI and the respective domains of total SDI were observed between patients receiving and not receiving vitamin D supplementation (disease-related SDI: 0.55 ± 0.94 vs 0.62 ± 0.95, *P* = 0.19) (S2 Table). Odds ratios for SDI and the respective domains of total SDI between the total enrolled and included patients receiving and not receiving vitamin D supplementation are shown in Fig 1. A significant association between diabetes and vitamin D supplementation was found in the sensitivity analysis (odds ratio: 0.46, 95% confidence interval [CI]: 0.22–0.99), but no significant association between the two was found after adjusting for confounding factors (age, sex, and current PSL dose) in the logistic regression analysis (odds ratio: 0.65, 95% CI: 0.28–1.40). Similarly, no significant differences in disease-related SDI and the respective domains of total SDI were observed between patients receiving and not receiving vitamin D supplementation in patients with disease duration ≥ 60 months (disease-related SDI: 0.61 ± 1.02 vs 0.70 ± 1.07, *P* = 0.33) or patients with prednisolone (disease-related SDI: 0.72 ± 1.13 vs 0.77 ± 1.13, *P* = 0.51).

ORs for SDI and the respective domains of total SDI (scores > 0) between the total enrolled and included patients receiving and not receiving vitamin D supplementation are compared. Closed circles represent the ORs, and bars represent the 95% CIs. GC, glucocorticoid; OR, odds ratio; SDI, SLICC/ACR Damage Index.

In both the total enrolled and included patients, no significant differences in SLEDAI scores were found between patients receiving and not receiving vitamin D supplementation (total enrolled patients: 5.5 ± 5.1 vs 5.2 ± 4.5, *P* = 0.62; included patients: 5.5 ± 4.9 vs 5.2 ± 4.5, *P* = 0.40).

## Discussion

In the present study, we described vitamin D supplementation status and evaluated the relationship between vitamin D supplementation and disease-related SDI scores associated with damage accumulation and disease activity in SLE patients. Approximately half of the patients received vitamin D supplementation, which is comparable to other observational study in Japan [29], and most of those had no history of fracture or avascular necrosis. Disease-related SDI did not statistically differ between patients receiving and not receiving vitamin D supplementation.

The clinical effects of vitamin D supplementation on disease activity control may be quite limited in SLE patients. Previous studies have reported no clinically significant effects of vitamin D supplementation on disease activity despite favourable immunological changes in SLE patients [30–33]. Only one randomised controlled trial reported the efficacy of vitamin D supplementation compared to placebo use in juvenile SLE patients [34]. However, in this study, immunosuppressive treatment in itself may have been insufficient because patients receiving vitamin D supplementation showed no improvement in SLEDAI scores. Because no significant clinical effects of vitamin D supplementation were observed on short-term disease activity control using sufficient immunosuppressive treatments, we evaluated disease-related SDI as a marker for long-term disease activity control and damage accumulation in SLE patients. However, no statistical association was observed between vitamin D supplementation and disease-related SDI. Therefore, we cannot conclude that there is enough evidence of effect of vitamin D supplementation on SLE, at least at the dosage indicated and prescribed in daily practice for osteoporosis, though the effect at higher dosages was not examined in this observational study.

Vitamin D supplementation should be considered for bone mineral metabolism despite its efficacy in disease activity control being unclear. In our study, vitamin D supplementation was prescribed frequently for younger patients with higher dose of PSL and fewer concomitant uses of bisphosphonates. A recent meta-analysis reported that higher-dose vitamin D supplementation could reduce fracture risk by 15%–29% [35]. SLE patients are at a high risk of osteoporosis and bone fractures [36], even if they have normal bone mineral density values [37]. Bone diseases in SLE patients are considered to be exclusively associated with damage caused by glucocorticoid use [19, 27, 28], and the ACR guidelines have recommended vitamin D supplementation for glucocorticoid-induced osteoporosis regardless of fracture risk [38]. In the present study, approximately half of the patients received vitamin D supplementation, which corresponds with data reported by several previous cohort studies [39–41]. Vitamin D supplementation in SLE patients has been considered to be safe and well tolerated, with reports of only mild or infrequent hypercalcaemia in previous studies [14, 42], and can be used for young women considering pregnancy for whom bisphosphonates were contraindicated. Vitamin D supplementation should be considered more proactively in all SLE patients, especially in young women without use of bisphosphonate.

This study had several limitations. First, we could not evaluate the dosing periods for vitamin D supplementation because of the cross-sectional design of the study. This may have led to an underestimation of the effects of vitamin D supplementation in SLE patients. However, medications for osteoporosis are generally administered long-term to prevent fractures rather than used for short periods of time in the acute phase. In practice, a retrospective study on prescriptions for osteoporosis in Japan reported a prescription continuation rate of more than 60% throughout the entire 18–24-month observation period [43]. Therefore, we may assume that the dosing periods in our study are long enough to evaluate. Second, there were no available data on vitamin D levels, calcium, phosphorus concentrations, and anti-phospholipids antibodies, which could have helped in examining the optimum dose for vitamin D supplementation. Insufficient doses of vitamin D supplementation and its sample size may also have led to an underestimation of its effects in SLE patients. Data on adherence was not collected in this study. However, it has been reported that drug adherence is better than 90% in SLE, even higher without harmful side effects [44]. So, the impact of adherence on the results may be relatively small. Also, our results might be less susceptible to vitamin D from other sources because supplement use of vitamin D and Vitamin D fortification of foods are uncommon in Japan [45]. Overall, despite these limitations, our large population study showed, if any, a limited impact of vitamin D supplementation on disease activity control in SLE patients in daily clinical practice.

## Conclusion

Even with a possible Vitamin D deficiency and a high risk of bone fractures in SLE patients, only half of our cohort received its supplementation. The effect of vitamin D supplementation for disease activity control was not observed.

## Supporting information

S1 Table Comparison of osteoporosis treatment status between patients with and without a history of fracture or avascular necrosis. (DOCX)

S2 Table Comparison of SDI scores and the respective domains of total SDI. (DOCX)
