# Supplementary material for: Real-world data on vitamin D supplementation and its impacts in systemic lupus erythematosus: Cross-sectional analysis of a lupus registry of nationwide institutions (LUNA)
Source: PLoS One. 2022 Jun 29;17(6):e0270569. doi: 10.1371/journal.pone.0270569 (PMC9242469; doi:10.1371/journal.pone.0270569)
Supplement: S1 Table — (DOCX) [file pone.0270569.s001.docx]

**Supplementary Table S1.** Comparison of osteoporosis treatment status between patients with and without a history of fracture or avascular necrosis

|  | Fractures/avascular necrosis (-)  (n = 775) | Fractures/avascular necrosis (+)  (n = 95) | *P* value |
| --- | --- | --- | --- |
| Mean age, years | 44.6 ± 14.5 | 51.3 ± 15.0 | <0.0001 |
| Number of female patients, n (%) | 693 (89) | 77 (81) | 0.02 |
| Mean PSL dose, mg/day | 7.2 ± 6.5 | 6.5 ± 5.9 | 0.30 |
| Vitamin D supplementation, n (%) | 377 (49) | 49 (52) | 0.59 |
| Calcium supplementation, n (%) | 29 (4) | 6 (6) | 0.23 |
| Bisphosphonate use, n (%) | 331 (43) | 38 (40) | 0.61 |
| Concomitant vitamin D supplementation and bisphosphonate use, n (%) | 144 (19) | 19 (20) | 0.74 |
| Teriparatide use, n (%) | 7 (1) | 7 (7) | <0.0001 |
| Denosumab use, n (%) | 9 (1) | 7 (7) | <0.0001 |
| Selective oestrogen receptor modulator use, n (%) | 4 (0.5) | 0 (0) | 0.48 |

*Values are expressed as mean ± standard deviation or number (percentage).

PSL, prednisolone.
