# Supplementary material for: Real-world data on vitamin D supplementation and its impacts in systemic lupus erythematosus: Cross-sectional analysis of a lupus registry of nationwide institutions (LUNA)
Source: PLoS One. 2022 Jun 29;17(6):e0270569. doi: 10.1371/journal.pone.0270569 (PMC9242469; doi:10.1371/journal.pone.0270569)
Supplement: S2 Table — (DOCX) [file pone.0270569.s002.docx]

**Supplementary Table S2.** Comparison of SDI scores and the respective domains of total SDI

|  | Primary analysis (n = 870) | | | Sensitivity analysis (n = 731) | | |
| --- | --- | --- | --- | --- | --- | --- |
|  | Vit. D (+)  (n = 426) | Vit. D (-)  (n = 444) | *P* value | Vit. D (+)  (n = 358) | Vit. D (-)  (n = 376) | *P* value |
| Total SDI | 1.10 ± 1.56 | 1.08 ± 1.40 | 0.56 | 0.70 ± 1.07 | 0.78 ± 1.09 | 0.22 |
| Disease-related SDI | 0.73 ± 1.16 | 0.73 ± 1.10 | 0.75 | 0.55 ± 0.94 | 0.62 ± 0.95 | 0.19 |
| Ocular | 0.20 ± 0.42 | 0.16 ± 0.36 | 0.19 | 0.14 ± 0.36 | 0.12 ± 0.32 | 0.47 |
| Neuropsychiatric | 0.13 ± 0.39 | 0.14 ± 0.38 | 0.58 | 0.12 ± 0.36 | 0.13 ± 0.37 | 0.66 |
| Renal | 0.16 ± 0.49 | 0.1 ± 0.51 | 0.44 | 0.08 ± 0.29 | 0.12 ± 0.34 | 0.09 |
| Pulmonary | 0.08 ± 0.29 | 0.06 ± 0.25 | 0.27 | 0.07 ± 0.27 | 0.06 ± 0.26 | 0.75 |
| Cardiovascular | 0.06 ± 0.25 | 0.05 ± 0.26 | 0.56 | 0.03 ± 0.19 | 0.03 ± 0.20 | 0.75 |
| Peripheral vascular | 0.07 ± 0.38 | 0.09 ± 0.42 | 0.16 | 0.05 ± 0.32 | 0.08 ± 0.37 | 0.06 |
| Gastrointestinal | 0.03 ± 0.21 | 0.03 ± 0.19 | 0.63 | 0.01 ± 0.14 | 0.03 ± 0.19 | 0.09 |
| Musculoskeletal | 0.22 ± 0.54 | 0.18 ± 0.53 | 0.19 | 0.06 ± 0.26 | 0.03 ± 0.18 | 0.15 |
| Skin | 0.07 ± 0.27 | 0.08 ± 0.32 | 0.90 | 0.07 ± 0.27 | 0.08 ± 0.32 | 0.99 |
| Gonadal failure | 0.04 ± 0.20 | 0.04 ± 0.19 | 0.64 | 0.03 ± 0.18 | 0.04 ± 0.20 | 0.65 |
| Diabetes | 0.04 ± 0.21 | 0.06 ± 0.24 | 0.29 | 0.03 ± 0.18 | 0.06 ± 0.23 | 0.13 |

*Values are expressed as mean ± standard deviation.

Vit., vitamin; SDI, SLICC/ACR Damage Index.
